# Massive Gene Loss and Function Shuffling in Appendicularians Stretch the Boundaries of Chordate Wnt Family Evolution

**DOI:** 10.3389/fcell.2021.700827

**Published:** 2021-06-09

**Authors:** Josep Martí-Solans, Hector Godoy-Marín, Miriam Diaz-Gracia, Takeshi A. Onuma, Hiroki Nishida, Ricard Albalat, Cristian Cañestro

**Affiliations:** ^1^Departament de Genètica, Microbiologia i Estadística, Institut de Recerca de la Biodiversitat (IRBio), Universitat de Barcelona, Barcelona, Spain; ^2^Department of Biological Sciences, Graduate School of Science, Osaka University, Osaka, Japan

**Keywords:** gene loss, gene function shuffling, chordate evolutionary developmental biology, wingless (Wnt) family evolution, appendicularian tunicate chordate

## Abstract

Gene loss is a pervasive source of genetic variation that influences species evolvability, biodiversity and the innovation of evolutionary adaptations. To better understand the evolutionary patterns and impact of gene loss, here we investigate as a case study the evolution of the wingless (Wnt) family in the appendicularian tunicate *Oikopleura dioica*, an emergent EvoDevo model characterized by its proneness to lose genes among chordates. Genome survey and phylogenetic analyses reveal that only four of the thirteen Wnt subfamilies have survived in *O. dioica*—Wnt5, Wnt10, Wnt11, and Wnt16,—representing the minimal Wnt repertoire described in chordates. While the loss of Wnt4 and Wnt8 likely occurred in the last common ancestor of tunicates, representing therefore a synapomorphy of this subphylum, the rest of losses occurred during the evolution of appendicularians. This work provides the first complete Wnt developmental expression atlas in a tunicate and the first insights into the evolution of Wnt developmental functions in appendicularians. Our work highlights three main evolutionary patterns of gene loss: (1) conservation of ancestral Wnt expression domains not affected by gene losses; (2) function shuffling among Wnt paralogs accompanied by gene losses; and (3) extinction of Wnt expression in certain embryonic directly correlated with gene losses. Overall our work reveals that in contrast to “conservative” pattern of evolution of cephalochordates and vertebrates, *O. dioica* shows an even more radical “liberal” evolutionary pattern than that described ascidian tunicates, stretching the boundaries of the malleability of Wnt family evolution in chordates.

## Introduction

The bloom of genomics is providing a new vision on gene loss as one of the major sources of genetic variation with great potential to contribute to evolutionary adaptation and the generation of biodiversity, and therefore to impact on the evolvability of groups of organisms (Olson, 1999; Albalat and Cañestro, 2016; Sharma et al., 2018; Guijarro-Clarke et al., 2020; Helsen et al., 2020; Xu and Guo, 2020). Despite mutational events that lead to non-functionalization and gene loss are random, the analyses of biased patterns of gene loss can reveal relevant information about the evolutionary impact of the losses (reviewed in Albalat and Cañestro, 2016). Thus, the loss of genes in the last common ancestor of a particular taxonomical group, or differences in the trends of gene loss of certain functional categories among different taxa might reflect differences in gene essentiality/dispensability resulting from differential selective restrictions or the evolution of divergent adaptations in different taxa. The wingless (*Wnt*) family, which encodes a set of secreted glycoprotein ligands that regulate key events of animal development, is a paradigmatic example of pervasive gene loss with different trends of gene loss among different taxa (Albalat and Cañestro, 2016). While deuterostomes, for instance, conserve in general most of the *Wnt* family repertoire, many protostomes have suffered extensive losses (Prud’homme et al., 2002; Darras et al., 2018; Somorjai et al., 2018). The loss of *Wnt3* in the last common ancestor of all protostomes can be considered a synapomorphic trait that might have impacted on the origin and evolution of this clade (Cho et al., 2010).

Gene loss, however, is not always adaptive, but in many occasions occurs under neutral conditions, as a consequence, for example, of a process of regressive evolution (reviewed in Albalat and Cañestro, 2016). Increase of mutational robustness or changes of environmental conditions can lead to an increase of the dispensability of certain genes, facilitating therefore selectively neutral gene loss without significant phenotypic impact (Albalat and Cañestro, 2016) as have been recently concluded after a comprehensive comparative genomic analysis across the metazoan tree of life (Fernández and Gabaldón, 2020). Gene losses have been frequently accompanied by events of function shuffling, in which paralogs or related gene families can co-opt redundant functions, and therefore increasing mutational robustness that can favor gene loss (McClintock et al., 2001; Cañestro et al., 2009). How function shuffling occurs, however, remains unclear. Promiscuous gene families, in which the activity of different paralogs is similar and functions among genes are interchangeable, are prone to bear function shuffling. Several examples of function shuffling accompanied by gene losses have been reported among Wnt genes (Somorjai et al., 2018) and references therein), which have been related to the function promiscuity of this gene family since multiple Wnt ligands can activate more than one pathway (Ring et al., 2014). *Wnt* ligands, after binding to the Frizzled receptor, can activate two different pathways: (i) the cell-fate pathway (a.k.a. “*Wnt*/β-catenin pathway” or the “canonical *Wnt* pathway”), which is mediated by the nuclear localization of β-catenin for pathway activation; and (ii) the cell-polarity pathway, which is mediated by several intermediate effectors acting independently of β-catenin, and includes, at least, the non-canonical planar cell polarity (PCP) pathway and the non-canonical *Wnt*/Calcium pathway (Loh et al., 2016).

Despite the Wnt family plays fundamental roles during development and adult tissue homeostasis in a vastly conserved way among metazoans, it still remains unclear how this gene family has evolved so radically different patterns of gene loss in different taxa, even within the same phylum. Within the chordate phylum, for instance, recent analysis of the *Wnt* family has revealed that cephalochordates show a “conservative” pattern of evolution, retaining the complete Wnt repertoire as single-member subfamilies (Somorjai et al., 2018). Vertebrates have maintained all the Wnt subfamilies, with the exception of WntA that has been associated to the evolution of an alternative mode to open a mouth, and have expanded the number of paralogs in Wnt subfamilies through the two rounds of whole genome duplication (Somorjai et al., 2018). Within tunicates, ascidians show a “liberal” pattern of evolution including ancestral synapomorphic gene losses (i.e., Wnt4 and Wnt8), some losses affecting specific ascidian lineages (e.g., Wnt1 in *Phelobobranchia* and Wnt3 in *Molgula*), and some burst of gene duplications affecting some ascidian groups (e.g., Wnt5 in *Stolidobranchia*). This liberal pattern of evolution has been argued that might have contributed to the morphological diversification of tunicates (Somorjai et al., 2018). In appendicularian tunicates, however, the evolution of the *Wnt* family remains unknown, and considering that these organisms are highly prone to lose genes (Ferrández-Roldán et al., 2019) and other signaling pathways such as retinoic acid has been dismantled (Martí-Solans et al., 2016), it appears as an attractive system to study the impact of gene loss and the limits of *Wnt* evolution in chordates.

To address this question, we have conducted an exhaustive survey of *Wnt* genes in genomic databases of *O. dioica* and have generated the first complete atlas of developmental expression of the Wnt family in appendicularians and the first fully described in all tunicates. Our study reveals a very dynamic evolution of Wnt signaling in *O. dioica* that would have led to an extraordinary reduction of the number of subfamilies—with only 4 out of the 13 subfamilies, which represents the smallest Wnt catalog described so far in chordates—accompanied by the expansion of the Wnt11 subfamily by lineage-specific gene duplications. Our detailed atlas of *Wnt* expression in *O. dioica* reveals that expression domains encompassed tissues derived from all three germ layers in a highly dynamic manner as well as several cases of “function shuffling.” Finally, our study also suggests that an asymmetrically localized maternal Wnt ligand is required for axis formation. Therefore, our results allow us to evaluate the contributions of different Wnt subfamilies during *O. dioica* development and to investigate the evolutionary and functional limits of *Wnt* signaling in chordate development.

## Materials and Methods

### Laboratory Culture of *Oikopleura dioica*

*O. dioica* specimens and embryos were obtained from animal colonies cultured in our lab for more than 5 years and originally collected in the Mediterranean coast of Barcelona (Catalonia, Spain) as previously described (Martí-Solans et al., 2015). Ovarian microinjection was performed as previously described (Omotezako et al., 2013; Supplementary Materials and Methods).

### Genome Database Searches and Phylogenetic Analyses

Protein sequences of the *Wnt* genes from vertebrate *Homo sapiens* and tunicate *Ciona robusta* were used as queries in BLASTp and tBLASTn searches in *O. dioica* genome databases^1^. Homologies were assigned by phylogenetic tree analyses based on Maximum Likelihood (ML) inferences calculated with PhyML v3.0 (Guindon et al., 2010) using protein alignments generated with MUSCLE and reviewed by hand (Edgar, 2004). Robustness of tree topologies was assessed under automatic model selection based on Akaike and Bayesian Information Criteria as well as by LG, WAG, and JTT substitution models. Due to computational load limitation of bootstrap performance, branch support was inferred by fast likelihood based methods aLRT SH-like and aBayes. Accession numbers for *O. dioica* sequences are provided in Supplementary Table 1.

### Gene Expression and Tissue Differentiation Analyses

Fragments of *O. dioica* genes were PCR amplified and cloned to synthesize gene-specific riboprobes (Supplementary Table 2). To reveal *Wnt* expression and evaluate neural tissues and notochord differentiation, whole-mount *in situ* hybridization on fixed embryos was performed as previously described (Bassham and Postlethwait, 2000; Cañestro and Postlethwait, 2007; Martí-Solans et al., 2016). Nuclear staining (1 μM Hoeschst in PBST for 1 h at room temperature) was included in expression analysis at tailbud stages to confirm muscle cell positions. α*-Tubulin A* and Brachyury were used as specific markers for neuronal tissues and notochord, respectively (Bassham and Postlethwait, 2000; Seo et al., 2004; Søviknes et al., 2007). Histochemical reaction of acetylcholinesterase (AChE) was used to examine the differentiation of muscle cells, while histochemical staining for alkaline phosphatase was used to monitor the differentiation of endoderm cells (Imai et al., 2000; Nishino et al., 2000; Omotezako et al., 2017). For germ-line differentiation, immunohistochemistry using an antibody against *Ciona robusta* Vasa homolog was carried out as previously reported (Onuma et al., 2017). The primary antibody used was affinity-purified rabbit anti-CiVH (1:500 dilution) (Shirae-Kurabayashi, 2006) and the secondary antibody used was Alexa Fluor 594 goat anti-rabbit IgG (1:500 dilution; Life Technologies).

## Results

### Only Four Wnt Subfamilies Have Survived in *O. dioica*

**FIGURE 1:**
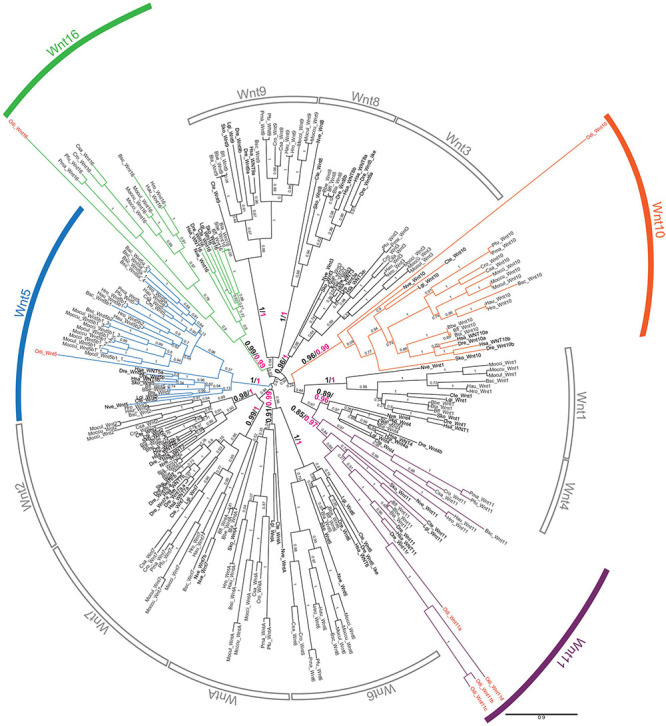
Phylogenetic analysis of the *Wnt* family. ML phylogenetic tree of the *Wnt* family in chordates reveals that *Wnt* gene repertoire in *Oikopleura dioica* (names in red) belongs to four subfamilies: Wnt 5 (blue), Wnt10 (orange), Wnt11 (purple), and Wnt16 (green). The scale bar indicates amino-acid substitutions. Node support values correspond to likelihood based methods aLRT SH-like (in black) and aBayes (in magenta); support of monophyletic Wnt subfamily nodes are indicated with greater fonts size. Species abbreviations: Chordate species: Tunicates: *Botryllus schlosseri* (Bsc), *Ciona savignyi* (Csa), *Ciona robusta* (Cro; formerly *Ciona intestinallis*), *Halocynthia roretzi* (Hro), *Halocynthia aurantium* (Hau), *Mogula occulta* (Moccu), *Mogula oculata* (Mocul), *Mogula occidentalis* (Mocci), *Phallusia fumigata* (Pfu) *Phallusia mammillata* (Pma), and *Oikopleura dioica* (Odi); Cephalochordates: *Branchiostoma belcheri* (*Bbe*), *Branchiostoma floridae* (*Bfl*), *Branchiostoma lanceolatum* (*Bla*); Vertebrates: *Danio rerio* (Dre), *Homo sapiens* (*Hsa*). Non-chordates species: hemichordate *Saccoglossus kowalevskii* (Sko), annelid *Capitella teleta* (Cte), mollusk *Lottia gigantea* (Lgi), and cnidarian *Nematostella vectensis* (Nve).

We conducted an exhaustive survey of *Wnt* genes in genomic database of *O. dioica* that revealed the presence of 8 putative *Wnt* sequences (Supplementary Table 1). To classify these *Wnt* genes, we performed phylogenetic reconstructions using a total of 254 *Wnt* sequences from 20 species representing all major metazoan groups, from the cnidarian *Nematostella vectensis* to the vertebrate *Homo sapiens.* The analysis recovered all 13 *Wnt* subfamilies as monophyletic groups and distributed *O. dioica Wnt*s among these 13 subfamilies with high support values (Figure 1). Maximum likelihood (ML) phylogenetic tree suggested that the 8 *O. dioica Wnts* belonged to 4 Wnt subfamilies—i.e., Wnt5, Wnt10, Wnt11 (5 sequences) and Wnt16 subfamilies—(Figure 1). The results, therefore, show that *O. dioica* have lost 9 Wnt subfamilies during its evolution. On the other hand, our results revealed that *O. dioica* has expanded the Wnt11 subfamily to at least 4 paralogs, named *Odi_Wnt11a* to *Odi_Wnt11d*. Analysis of the gene structure of the *Odi_Wnt11* paralogs showed that *Odi_Wnt11a* had 5 introns, one of them in a conserved position in all *Wnt* genes across all metazoans (boxed black arrowhead in Supplementary Figure 1A), and other intron located in a position considered a signature of Wnt11 subfamily in bilaterians (Cho et al., 2010; green arrowhead in Supplementary Figure 1), further supporting its orthology. The other 3 *Odi_Wnt11* paralogs, namely *Odi_Wnt11b, Odi_Wnt11c*, and *Odi_Wnt11d*, had no introns, pointing to the possibility of a retrotranscriptional origin during the evolution of the appendicularian lineage. Our genome survey found a partial sequence of a potential duplicate of Wnt11c with a similarity > 94% in the genome assemblies of *O. dioica* specimens from Norway (Wnt11c_NOR2: GSOIDG00009921001) as well as from Osaka (Wnt11c_OSA2: OSKA2016.S19.g13171.01) that may have been independently duplicated in both populations (Supplementary Figure 1B). However, the fact that no expression of this potential Wnt11c duplicate was detected in the gene expression matrix of the Oikobase (Supplementary Figure 6) nor in any of the ESTs collections of any of the two populations, together with the fact that this duplicated Wnt11c could not be amplified by PCR on genomic DNA nor cDNA from specimens from Barcelona, suggested that these could be allelic variants that have been artifactually duplicated during the genome assemblies, rather than actual gene duplicates. Whole genome sequencing with a telomere-to-telomere quality from specimens of Barcelona and other locations, and further interpopulation comparisons will be needed to clarify if *Wnt11c* has been independently duplicated in different *O. dioica* populations or these sequences simply correspond to allelic variants of *Wnt11c*.

The Wnt signaling pathway, far from simple, depends on the action of multiple genes (i.e., Wnt receptors, secreted Wnt inhibitors, intermediate effectors, etc.), which we wondered if they could have been also affected by the massive loss of *Wnt* ligands in *O. dioica*. To investigate this possibility and to assess the conservation of the Wnt signaling pathways in *O. dioica*, we used the KEGG Automatic Annotation Server (KAAS) (Moriya et al., 2007) on *O. dioica* genomic and transcriptomic data from Danks et al. (2013) to automatically identify the *O. dioica* orthologs to the components of the three main Wnt signaling pathways. KAAS analysis revealed conservation of the key components of the three Wnt pathways, with the exception of Axin, APC and several antagonists (Supplementary Figure 2A and Supplementary Table 1). Phylogenetic analysis in *O. dioica* of Wnt receptor *Frizzled* (*Fzd*) (Supplementary Figure 2B) and Wnt antagonist *secreted Frizzled related protein* (*sFRP*) (Supplementary Figure 2C) revealed, in addition, a decrease in the diversity of these components in this species. *O. dioica* appeared to have lost 2 out of the 5 Fzd subfamilies present in ascidians and vertebrates –retaining members in the Fdz1/2/7 (1 gene), Fdz3/6 (3 genes) and Fdz5/8 (1 gene) subfamilies– and had only 1 sFRP (*Odi_sFRP2*), in contrast to the 5 and 3 sFRP subfamilies found in vertebrates and ascidians, respectively.

### Wnts Are Expressed in All Germ-Layers During *O. dioica* Development

**FIGURE 2:**
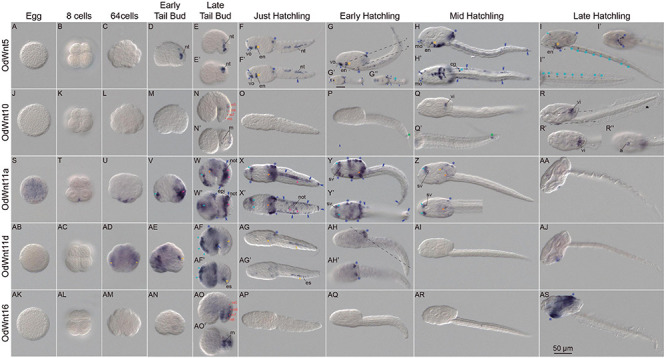
Expression patterns of *Oikopleura dioica Wnt* genes during development. Whole-mount *in situ* hybridization in *O. dioica* eggs **(A,J,S,AB,AK)**, 8 cell embryos **(B,K,T,AC,AL)**, 64 cell embryos **(C,L,U,AD,AM)**, early tail bud embryos **(D,M,V,AE,AN)**, late tail bud embryos **(E,N,W,A,F,AO)**, just hatchlings **(F,O,X,AG,AP)**, early hatchlings **(G,P,Y,AH,AQ)**, mid hatchlings **(H,Q,Z,AI,AR)** and late hatchlings **(I,R,AA,AJ,AS)**. Upper image of each panel corresponds to left lateral view oriented anterior toward the left and dorsal toward the top. Bottom images (‘) are ventral views of optical cross sections at the levels of dashed lines. Red dots label muscle cell nuclei; magenta arrowheads label notochord; orange arrowheads label mesoendoderm; yellow arrowheads label endodermal strand; yellow asterisks label endostyle; light blue arrowheads label neuroectoderm and anterior brain; blue light asterisks label neural tube; green asterisk labels a cell posterior to the tip of the notochord that according to tubuline expression could be of neural condition. Blue dark arrowheads label sensory epidermis; blue dark asterisks label oikoplastic epithelium; blue dark double arrowheads label caudal epithelium and blue dark arrow labels posterior trunk epidermis. a, anus; cg, caudal ganglion; en, endostyle; epi, epidermis; es, endodermal strand, m, muscle cell pair; mo, mouth; not, notochord; nt, neural tube; sv, sensory vesicle; vi, vertical intestine; vo, ventral organ. Scale bar = 50.

To explore the functional consequences of the *Wnt* losses and duplications in *O. dioica* developmental programs, we investigated the expression patterns of all *O. dioica Wnt* genes during embryogenesis and larval development by whole-mount *in situ* hybridizations (WISH). Results revealed that 5 out the identified *Wnt* genes showed complex tissue-specific expression patterns that changed throughout different developmental stages (Figure 2), whereas no signal was detected for two *Wnt11* paralogs, namely *Odi_Wnt11b* and *Odi_Wnt11c* (Supplementary Figure 3), suggesting that they may function at adult stages, or they were integrated after retrotranscription in a genomic region in which no transcription is promoted.

Because many *Wnt* genes are maternally expressed and play a role in establishing the primary axis in several metazoan species, we paid special attention to the *Wnt* expression in *O. dioica* eggs. WISH results revealed that from all *Wnt* genes only *Odi_Wnt11a* was part of the maternal component (Figure 2S). This *Wnt* paralog was, therefore, the best candidate to participate in establishing primary embryonic axis in *O. dioica* (see section following for details).

All the other *Wnt* appeared to only have zygotic expression. Of these other *Wnt*, *Odi_Wnt11d* signal was the first detected at approximately 64-cell stage. *Odi_Wnt11d* signal was detected in two internal domains, which could be endodermal derivates precursors, such as endostyle and endodermal strand (Figure 2AD yellow dots), while *Odi_Wnt11a* began its zygotic expression in several blastomeres in the vegetal part of the embryo (Figure 2U). The onset of *Odi_Wnt5* and *Odi_Wnt10* and *Odi_Wnt16* expressions occurred later, at tailbud stages, when new expression domains appeared in a highly dynamic fashion encompassing tissues derived from all three germ layers.

In the mesoderm, *Wnt* expression signal was observed in a limited number of cells of the musculature and notochord. Thus, *Odi_Wnt10* appeared to be expressed in the 5th muscle cell pair of late tailbuds (m5 in Figure 2N), while *Odi_Wnt16* signal appeared in the 6, 7, and 8th muscle cell pairs (m6-8 in Figure 2AO). At this stage, *Odi_Wnt11a* signal appeared to be restricted to the posterior third of the notochord (Figures 2V–X magenta arrowheads) and to the meso/endoderm in the posterior part of the trunk, anteriorly to the first cell of the notochord (Figure 2W orange arrowheads). This meso/endodermal expression was maintained until mid-hatchling stages, when traces of *Odi_Wnt11a* signal could be still detected in the trunk (Figures 2X–Z orange arrowheads).

In the endoderm, we also detected *Wnt* signal domains. From early tailbud to just-hatchling stages, *Wnt11d* labeled a group of posterior cells of the tail at the right side of notochord that correspond to the region populated by the endodermal strand (Figures 2AE–AF yellow arrowheads and Supplementary Figures 4C,D). Later, *Odi_Wnt11d* signal was detected in a region of the posterior part of the trunk where presumably the endodermal strand cells migrate (Figure 2AG yellow arrowheads and Supplementary Figure 4E). After hatch, the endostyle primordium showed a temporal expression of *Odi_Wnt11d* and *Odi_Wnt5* that was maintained up to late hatchling stages restricted to the most ventral and posterior part of the organ (Figures 2F–I yellow asterisk and Supplementary Figure 4E). At mid-hatchling stage, part of the stomach primordium was labeled by *Odi_Wnt10*, an expression domain that was maintained up to late-hatchling stages mostly restricted to the vertical intestine, to the most external part of the rectum in the opening of the anus, and to the connection between the esophagus and left stomach (Figures 2Q,R yellow arrowheads and Supplementary Figure 4A). At mid-hatchling stages, *Odi_Wnt11a* showed a faint expression in the connection between the two stomach lobes (Figure 2Z yellow arrowhead).

We also observed *Wnt* expression signal in derivatives of the ectoderm, including the nervous system and epidermis. At tailbud stage, *Odi_Wnt11a* labeled an anterior region of the trunk, in the presumptive area where the pharynx and the anterior brain will develop (Figure 2V light blue arrowheads). This expression persisted until the mid-hatchling stage, in which signal appeared labeling two bilateral domains, one adjacent to the sensory vesicle and the other at the outer part of the brain near to the epidermis, plausibly corresponding to the dorsal nerve secretomotor neurons (Olsson et al., 1990; Figures 2W–Z light blue arrowheads). Also, at tailbud stages, *Odi_Wnt11d* signal was observed in the neuroectoderm dorsally located to the brain, which faded in posterior stages (Figure 2AF light blue arrowheads), while *Odi_Wnt5* signal was observed in the posterior part of the tail, likely corresponding to the developing neural tube (Figures 2D,E). By hatchling stages, *Odi_Wnt5* signal exhibited a scattered pattern throughout the neural tube including the caudal ganglion (Figures 2F–H light blue asterisks), which became evenly distributed in late hatchlings reflecting the distribution of neurons throughout the nerve tube. *Odi_Wnt5* signal was also observed in the anterior brain of late hatchlings (Figure 2I light blue asterisks and arrowheads, respectively). Besides, *Odi_Wnt10* signal was also detected in nervous system, specifically in the caudal ganglion by late tailbud stage (Supplementary Figure 4A light blue asterisk). From early hatchling to mid hatchling stages, *Odi_Wnt10* also labeled a single cell posterior to the end of the notochord (Figures 2P,Q green asterisk). This position of this conspicuous cell could correspond with an α*-tubulin* positive cell described during the characterization of the nervous system in *O. diocia* (Søviknes et al., 2007), although the ontogenic nature of this cell needs further investigation (Onuma et al., 2020).

Regarding the epidermis, *Wnt* signal was observed in four different types of epidermal domains. (i) Domains in the trunk epidermis related to sensory or placode-related cells connected to the nervous system. These domains included the ventral organ and the lateral of the mouth for *Odi_Wnt5* signal (Figures 2F–H dark blue arrowheads), and the paired Langerhans receptor primordia in the posterior part of the trunk for *Odi_Wnt11a* signal (Figures 2W–Y dark blue arrowheads). (ii) Domains in the oikoplastic epithelium. The mid-dorsal domain of the oikoplast was labeled by *Odi_Wnt11a* (Figures 2X–Z), which also labeled the posterior-dorsal domain together with *Odi_Wnt5, Odi_Wnt11d*, and *Odi_Wnt16* (Figures 2F–I, **AF–AH, AS** dark blue asterisks) and the ventral oikoplast, along with *Odi_Wnt5* and *Odi_Wnt16* (Figures 2G-I,X-Y,AS and Supplementary Figure 4B dark blue asterisks). (iii) A domain in the lateral of the tail. A cell band in the equator of the tail together with the most distal epidermal cell of the tail was labeled by *Odi_Wnt5* and *Odi_Wnt11a* (Figures 2F–H,W–Y, dark blue double arrowheads). And iv) a domain in the posterior-ventral epidermis of the trunk, close to the separation between the trunk and the tail, was labeled by *Odi_Wnt11d* (Figure 2AF dark blue arrow).

### Maternal Odi_Wnt11a Transcripts Are Asymmetrically Localized in the Posterior-Vegetal Region of Cleaving Embryos

**FIGURE 3:**
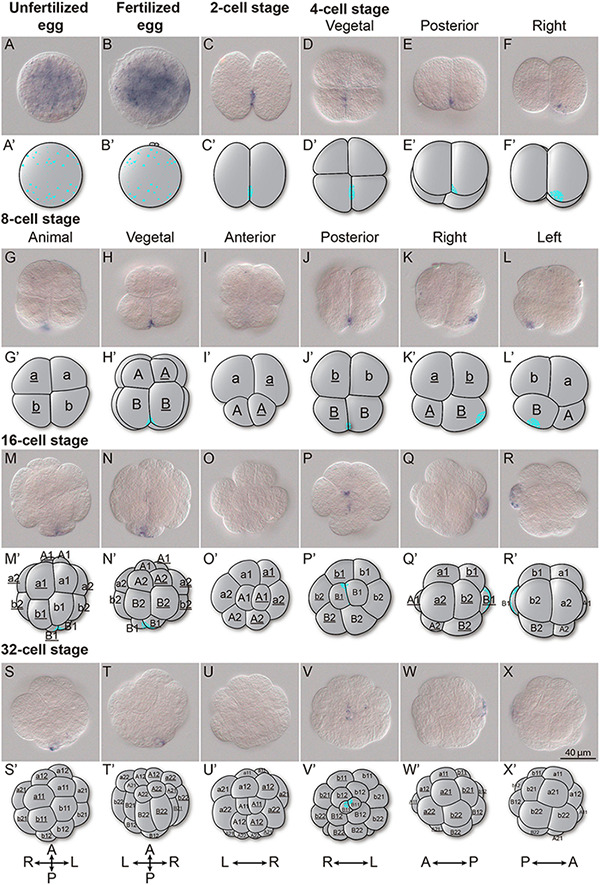
*Wnt11a* expression in cleavage stages. Whole-mount *in situ* hybridization in *O. dioica* from egg up to the 32-cell stage revealing an asymmetric localization of the *Wnt11a* maternal transcripts. **(A)** Unfertilized egg. **(B)** Fertilized egg. **(C)** 2 cells stage. **(D–F)** 4 cells stage. **(G–L)** 8 cells stage. **(M–R)** 16 cells stage. **(S–X)** 32 cells stage. Schematic representations adapted form (Fujii et al., 2008) and blastomere names according to Delsman’s nomenclature are given. Embryos were viewed from various directions indicated at the top. Scale bar = 40 μm.

As mentioned above, *Odi_Wnt11a* appeared as the only *Wnt* gene maternally transcribed in *O. dioica* (Figure 2S). Detail analyses during the first five cleavages of *O. dioica* embryos revealed that *Odi_Wnt11a* transcripts became asymmetrically localized in the two most posterior-vegetal cells (Figure 3). In eggs, both before and after fertilization, *Odi_Wnt11a* signal was uniformly distributed throughout the cytoplasm (Figures 3A,B). After the first division, however, *Odi_Wnt11a* signal appeared mostly accumulated nearby the contact region of the cell membranes of the two daughter cells, in the prospective vegetal-posterior pole of the embryo (Figure 3C). After the second division, the signal was only detected in the two posterior cells, only visible in the area toward the presumptive vegetal pole (Figures 3D–F). After the third division, at 8 cell-stage, *Odi_Wnt11a* signal continued restricted to the posterior region of a pair of posterior vegetal blastomeres, named B/B according to Delsman’s nomenclature (Delsman, 1910; Figures 3G–L), equivalent to B4.1/B4.1 blastomeres according to the ascidian nomenclature system (to facilitate comparisons, ascidian nomenclature is indicated in parentheses) (Conklin, 1905; Stach et al., 2008). After the fourth and fifth divisions, *Odi_Wnt11a* signal remained in the blastomere pairs B1/B1 (B5.2/B5.2) and B11/B11 (B6.4/B6.4), which could be recognized by being the smallest ones for each division (Figures 3M–X). According to the fate map of *O. dioica*, these blastomeres are internalized during gastrulation, remain cleavage-arrested until the hatchling stage, and plausibly become the primordial germ cells (Fujii et al., 2008; Stach et al., 2008; Olsen et al., 2018).

### Odi_Wnt11a *Knockdown*

**FIGURE 4:**
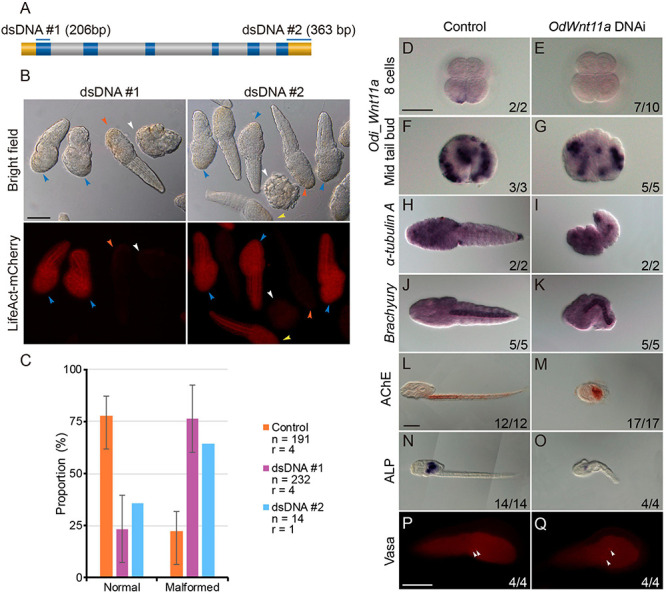
*Wnt11a* knockdown phenotypes. **(A)** Regions targeted by each of the PCR products. Upper box is a gene model of *Wnt11a* and the regions targeted by each of the PCR products are shown by blue bars above. Blue, gray and yellow boxes indicate the ORF, intron and UTR, respectively. **(B)** Embryos co-injection with *Wnt11a* PCR products (0.2 μg/μl) and Lifeact-mCherry mRNA (0.7 μg/μl). Only *mCherry* positive embryos were examined. Phenotypes were categorized into two groups: normal and short. Two-thirds of the animals with fluorescence show shortened trunk and tail (blue arrowheads), although some animals with fluorescence did not show malformations (yellow arrowheads). Most of the animals without fluorescence developed normally (vermillion arrowheads), while some embryos without fluorescence underwent developmental arrested before hatching (white arrowhead). **(C)** Proportions of each of the phenotype of hatched larvae after injection with the three kinds of PCR products. Orange, purple, and blue bars show the result of Control (*Kaede* PCR product), DNAi #1 and DNAi #2, respectively. Number of analyzed embryos (n) and number replicas (r) are indicated in the legend. **(D–G)** Whole-mount *in situ* hybridization for *Wnt11a* in 8 cells embryos **(D,E)** and mid tail bud stage **(F,G)** injected with *Kaede* PCR product as control and *Odi_Wnt11a* DNAi #1 PCR product. **(H,I)** Whole-mount *in situ* hybridization for the neuronal marker α*-Tubulin A* in just hatchlings injected with *Kaede* PCR product and *Odi_Wnt11a* DNAi #1 PCR product. **(J,K)** Whole-mount *in situ* hybridization for the notochord marker *Brachyur*y in just hatchlings injected with *Kaede* PCR product and *Odi_Wnt11a* DNAi #1 PCR product. L and M. Histochemical staining for the muscle differentiation marker acetylcholinesterase (AChE) in larvae injected with *Kaede* PCR product and *Odi_Wnt11a* DNAi #1 PCR product. **(N,O)** Histochemical staining for the endoderm differentiation marker alkaline phosphatase (ALP) in larvae injected with *Kaede* PCR product and *Odi_Wnt11a* DNAi #1 PCR product. **(P,Q)** Immunohistochemistry for the germ line marker vasa in just hatchlings injected with *Kaede* PCR product and *Odi_Wnt11a* DNAi #1 PCR product. The ratio, at the bottom of each panel, indicates the proportion of embryos with the phenotype shown. Scale bar = 50 μm.

Among the seven *Wnt* genes of *O. dioica*, we focused on *Odi_Wnt11a* because the asymmetrical distribution of its maternal component suggested a possible role in the establishment of the embryonic primary axis. To investigate the function *Odi_Wnt11a* during development, we generated knockdown animals using a DNAi approach (Omotezako et al., 2017, 2015), which consisted in the microinjection of a double strand DNA (dsDNA) against the target gene. We PCR amplified two dsDNA fragments of 206 bp and 363 bp that extended over the first exon and the 3’UTR region of the *Odi_Wnt11a*, respectively (Figure 4A and Supplementary Figure 5). These dsDNA were co-injected with an mRNA encoding for *Lifeact*-mCherry fusion protein into the ovary of pre-spawning females (Omotezako et al., 2013;see section Materials and Methods in Supporting Information for a detail description of dsDNA technique). As expected from this experimental approach, a gradient of dsDNA and mCherry was generated in the syncytial gonad from the point of injection. In the clutch from an injected female we found, therefore, both fluorescent mCherry embryos (Figure 4B blue and yellow arrowheads) and non-fluorescent embryos (Figure 4B vermillion and white arrowheads). According to previous works (Omotezako et al., 2015), we assumed that fluorescent embryos had incorporated the dsDNA, and therefore could show an altered phenotype, whereas non-fluorescent embryos should develop normally. The analysis of the phenotypes of animals from clutches of injected females with both dsDNA against *Odi_Wnt11a* showed that more than two thirds of *mCherry*-positive larvae showed similar abnormal morphologies (trunks and tails shorter than non-fluorescent siblings; Figures 4B,C), whereas control animals injected with a mock dsDNA (against Kaede protein) did not show such malformations. These results supported the specificity and reproducibility of the phenotype caused by both *Odi_Wnt11a* dsDNA. *In situ* hybridization of *Odi_Wnt11a* revealed a strong reduction *Wnt* signal in the posterior vegetal blastomeres (B/B) of 8-cell fluorescent embryos (Figures 4D,E). This reduction was, however, less strong in mid tailbud stage embryos (Figures 4F,G), which suggested that the dsDNA injected in the ovary was not able to completely silence zygotic transcription.

To understand the functional consequences in the *O. dioica* development of knockdown *Odi_Wnt11a* by dsDNA injection, we performed WISH and immunohistochemical analyses as well as enzymatic activity assays with several tissue-specific developmental markers, including those for neural tissue, notochord, muscle, endoderm or germ line. α*-Tubulin A* has been established as a general neuronal marker with expression in brain nerves, cerebral and caudal ganglia and nerve cord in *O. dioica* larvae (Seo et al., 2004; Søviknes et al., 2007). WISH analysis of α*-Tubulin A* showed similar expression level in the neurons of the trunk and tail when malformed (knockdown) and normal (control) larvae were compared (Figures 4H,I). We next analyzed the expression of *brachyury*, as a notochord marker, and the activity of acetylcholinesterase (AchE), as a muscle marker (Bassham and Postlethwait, 2000; Nishino et al., 2000; Omotezako et al., 2017), to evaluate the affectation of mesodermal tissues. Results showed that despite the malformations of the embryos, both notochord and muscle cells were present in knockdown larvae (Figures 4J–M). Endodermal cells were visualized in the inner region of the *O. dioica* trunk labeling the digestive tract by the activity of alkaline phosphatase (ALP) (Imai et al., 2000; Omotezako et al., 2017). ALP activity was detected in knockdown larvae, although the extension and intensity of the signal was clearly reduced (Figures 4N,O). Finally, because B11/B11 blastomeres, in which *Odi_Wnt11a* is asymmetrically located, are the precursor cells of the primordial germ cells (PGC), we investigated whether knocking down *Odi_Wnt11a* might affect PGC determination. We analyzed the expression of *vasa*, a germ-line specific marker (Ganot et al., 2007; Henriet et al., 2015; Olsen et al., 2018). Immunohistochemistry with an ascidian vasa anti-body (Shirae-Kurabayashi, 2006), labeled two cells in the posterior part of the trunk (Figures 4P,Q) in both, treated and control larvae, suggesting that maternal *Odi_Wnt11a* was not necessary for PGC determination.

In summary, the analysis of different cell- and tissue-specific markers indicated that knockdown maternal *Odi_Wnt11a* caused major morphological malformations such as shorter trunks and tails with bended notochords affecting tail elongation, as well as some impaired endodermal structures, but it did not seem to affect germ-layer specification neither overall tissue differentiation. Further investigations will be needed to establish whether maternal *Odi_Wnt11a* was not relevant for axial developmental processes or whether zygotic expression *Odi_Wnt11a*, which was only slightly affected by dsDNA injections, might be functionally compensating the maternal component.

## Discussion

### Evolution of the Wnt Repertoire in *O. dioica*

**FIGURE 5:**
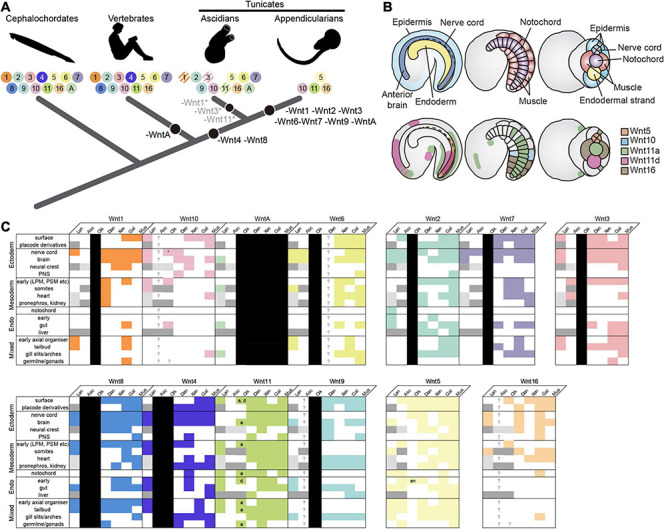
Evolutionary comparison of the Wnt gene repertoire and expression domains between *Oikopleura dioica* and other chordates. **(A)** Evolution of the Wnt subfamily repertoire (colored circles with numbers) highlighting Wnt losses (black circles) across the chordate evolutionary tree. *O. dioica*, with only 4 Wnt gene families—*Wnt5*, *Wnt10*, *Wnt11*, and *Wnt16*–, represents the minimal *Wnt* repertoire among all chordates analyzed so far. Striped circles indicate gene losses affecting only specific groups or species (e.g., Wnt1 loss in Phlebobranchia, *Wnt11* in Molgulas, and *Wnt3* in *Botryllus*). **(B)** Schematic illustration of *Wnt* expression in *O. dioica*. Upper panel shows drawings of *O. dioica* mid tailbud embryo in lateral view (left and center) and ventral view (right), with the position of central nervous system (CNS, dark blue), epidermis (light blue), endoderm (yellow), notochord (purple), and muscles (red) depicted. Lower panels show the expression of *O. dioica*’s Wnt genes in the mid tailbud stage. **(C)** Filled colored boxes denote documented expression in Somorjai et al. (2018); white filled boxes indicate absence of, or unreported expression; question marks are reserved for genes that are present, but whose expression has never been assessed; black boxes represent lost genes; dark gray boxes denote tissues/structures that are absent in the subphylum and/or at examined stages, and stippled gray boxes highlight tissues/structures lacking clear homologs, in which therefore, expression comparison is not possible. Expression patterns for vertebrate paralogs of the same Wnt subfamily were combined. *O. dioica’*s paralogs are indicated (*a* = Odi_Wnt11a; *d* = Odi_Wnt11d). Asterisk: caudal ganglion expression (see Supplementary Figure 4). Lan, lancelet; Asc, ascidian; Oik, *O. dioica*; Dan, zebrafish *Danio rerio*; Xen, frog *Xenopus laevis/tropicalis*; Gal, chicken *Gallus gallus*; Mus, mouse *Mus musculus*; PNS (ectoderm), peripheral nervous system; LPM/PSM (mesoderm), lateral plate/presomitic mesoderm; en, endostyle.

The repertoire of *Wnt* ligands in deuterostomes –ambulacraria plus chordates– seems, in general, refractory to gene loss. In ambulacraria, for instance, the hemichordate *Saccoglossus kowalevskii* has retained the complete set of Wnt subfamilies (Darras et al., 2018), while echinoderms have all the Wnt subfamilies except the Wnt11 (Croce et al., 2006; McCauley et al., 2013; Robert et al., 2014). Similarly, two of the three chordate subphyla –cephalochordates and vertebrates– also follow this “conservative” pattern of Wnt evolution preserving all Wnt subfamilies, with the exception of WntA in vertebrates, and the duplication of several Wnt subfamilies as a product of the two rounds of whole genome duplication occurred at the base of the vertebrates (Somorjai et al., 2018). Our previous work on ascidian *Wnt* repertoire (Somorjai et al., 2018), and specially our present analysis in *O. dioica* indicates that, in contrast to all other chordates, tunicates have adopted a “liberal” pattern of evolution including several gene losses and duplications. According to our phylogenetic reconstructions, Wnt4 and Wnt8 subfamilies were lost in both ascidian and appendicularian species, and therefore, likely due to an early loss event in the last common ancestor of all tunicates. The ancestral loss of Wnt4 and Wnt8, therefore, appears as a synapomorphic trait that might have differentially influenced the evolutionary divergence of tunicates in contrast to other chordates (Figure 5A). In addition, the independent loss of numerous Wnt subfamilies in some groups of ascidians (i.e., Wnt1, Wnt3, and Wnt11) and in *O. dioica* (i.e., Wnt1, Wnt2, Wnt3, Wnt6, Wnt7, Wnt9, and WntA subfamilies), some of them in parallel in different tunicate lineages (i.e., Wnt1, Wnt3) suggest that the selective competence to lose Wnt genes differ between tunicates and the other chordates. *O. dioica*, which has retained members in only 4 Wnt subfamilies (i.e., Wnt5, Wnt10, Wnt11, and Wnt16; Figure 5A) appears as the extreme case pushing the limits of reducing the Wnt repertoire in chordates. *O. dioica*, together with the protostome gastropod *Patella vulgata* with also only 4 Wnt gene families (Prud’homme et al., 2002), would represent the minimal Wnt repertoire among all bilaterians analyzed so far.

The loss of Wnt subfamilies during the evolution of the appendicularian lineage has been accompanied by the expansion of the Wnt11 subfamily up to 4 paralogs (*Odi_Wnt11a*, *Odi_Wnt11b*, *Odi_Wnt11c*, and *Odi_Wnt11d*). The intronless structure of *Odi_Wnt11b*, *Odi_Wnt11c*, and *Odi_Wnt11d* suggest a retrotranscriptional origin of these genes from an ancestral *Odi_Wnt11a* that still retain introns (Supplementary Figure 1; Kaessmann et al., 2009). Often, intronless retroposed gene copies have been viewed as evolutionary dead-ends with little biological relevance due to the lack of regulatory elements. Although this may be case for *Wnt11b* and *Wnt11c* (Supplementary Figures 3, 6), *Wnt11d* clearly showed a specific and dynamic expression pattern (Figures 2AD–AH), suggesting that it has achieved a biological role and has recruited regulatory elements that drive its expression.

Interestingly, the reduction in the repertoire of Wnt subfamilies has been accompanied by a reduction in the number of subfamilies of Wnt receptors and of antagonists (Supplementary Figures 2B,C), which may suggest a possible parallel gene elimination (or gene coelimination) in the Wnt activators repertoire (i.e., *Wnt* ligands and receptors) (Supplementary Figure 2). The conservation and expression of most of the intermediate effectors and nuclear effectors (Supplementary Figures 2A, 6 and Supplementary Table 1) would indicate that the signaling pathway is totally functional in this species despite the reduction, and it will require further investigations to see how the loss of Wnt subfamilies have influenced the evolution of downstream signaling effectors.

### Comparative Analysis of Wnt Expression During Embryogenesis of *O. dioica* and Other Chordates

It is generally accepted in EvoDevo that orthologous genes in the same subfamily often play conserved functions across evolutionarily distant species (Dolinski and Botstein, 2007). The comparison of the expression patterns of *O. dioica Wnt* genes with other chordate species further support this general notion (Figures 5B,C). *O. dioica*, however, has lost many Wnt subfamilies, and some of these losses have been possible because these subfamilies have become, somehow, dispensable. Our analysis of *O. dioica* has revealed that Wnt dispensability might be associated to synfunctionalization events (Gitelman, 2007)—i.e., one paralog acquires the expression domain of another, replacing it—leading to function shuffling when different lineages are compared (McClintock et al., 2001), or caused when a Wnt subfamily has become non-essential for *O. dioica* development. Our study of *O. dioica* Wnt genes reveals, therefore, examples of three evolutionary patterns: patterns of functional conservation, patterns of functional shuffling, and patterns of functional extinction.

Regarding the first evolutionary pattern, expression analyses suggest that many *O. dioica* Wnt orthologs conserve ancestral expression domains related to their functions in homologous structures. For instance, *Wnt11* is expressed in endodermal derivates in cephalochordates and vertebrates (Schubert et al., 2000a; Sinner et al., 2006; Cui et al., 2011; Somorjai et al., 2018), and in O. dioica *O*di_*Wnt11d* is expressed in the endostyle and the endodermal strand (Figures 2AE–AG, 5B,C and Supplementary Figure 4D), suggesting a functional conservation of Wnt11 endodermal signaling in chordates. Similarly, *O*di_*Wnt11a* is expressed in the posterior part of the notochord (Figures 2V–X, 5B), likewise *Wnt11* is expressed in the notochord of cephalochordates and vertebrates (Makita et al., 1998; Sasakura et al., 1998; Baranski et al., 2000; Schubert et al., 2001; Andre et al., 2015; Figure 5C). Besides, it has been shown that down-regulation of *Wnt11* expression produces miss elongation of the A-P axis in vertebrates (Rauch et al., 1997; Yamaguchi et al., 1999; Heisenberg et al., 2000; Tada and Smith, 2000; Nakamura et al., 2006; Niwano et al., 2009). Similarly, knocking-down *Odi_Wnt11a* produces *O. dioica* larvae with shortened trunks and tails. It can be argued, therefore, that the ancestral chordate function of *Wnt11* in endoderm and notochord has been preserved in *O. dioica*, although subfunctionalized between paralogs, that is, between endodermal *Odi*_*Wnt11d* and notochordal *Odi_Wnt11a*.

Regarding the second evolutionary pattern, synfunctionalization of *O. dioica Wnt* genes might appear as function shuffling events when compared with other chordate species. For example, *O. dioica* has lost Wnt6 *and* Wnt7 subfamilies (Figure 5A), which are expressed in the neural crest (NC) and the central nervous system (CNS) in all other chordates (Sasakura and Makabe, 2000; Schubert et al., 2001, 2000b; García-Castro et al., 2002; Imai et al., 2004; Martin et al., n.d.). *Odi_Wnt5* appears to have synfunctionalized in the *O. dioica* lineage compensating for Wnt6 and Wnt7 losses as it is expressed in the nerve cord during *O. dioica* embryogenesis (Figures 5B,C) (notice that *Wnt5* is not involved in the development and differentiation of the neuronal system in cephalochordates (Schubert et al., 2001), neither is expressed in the neural tube of ascidians (Miya and Nishida, 2002; Imai et al., 2004) (although the ascidian *Halocynthia roretzi* seems to transiently express *Wnt5*α in blastomeres A8.15/16 precursors of the spinal cord; Sasakura et al., 1998). Function shuffling among *Wnt5* and *Wnt6* or *Wnt7* has therefore occurred during the evolution of different chordate lineages. Function shuffling is also observed when comparing the *Wnt* genes responsible for determination of primary body axis. Whereas *Odi_Wnt11a* is the main candidate for this role in *O. dioica* (Figure 3), *Wnt5* in ascidians (Sasakura et al., 1998; Imai et al., 2004), *Wnt8* in zebrafish (Lu et al., 2011), and *Wnt11* in *Xenopus* (Tao et al., 2005) have been associated with this function in these species.

Regarding the third evolutionary pattern, the development of a structure that is Wnt-dependent in chordates appears to have become Wnt-independent in *O. dioica*, leading to the loss of *Wnt* genes. For example, the formation of the gill slits appears to be independent of Wnt signaling in *O. dioica* (i.e., none of the *O. dioica Wnt* genes were expressed in the gill slits), whereas Wnt signaling (together with retinoic acid signaling) appears necessary for the formation of the homologous structures in amphioxus [*Wnt9* and *WntA* are expressed in the gill arches (Onai et al., 2009; Somorjai et al., 2018) and vertebrates (*Wnt2, Wnt4, Wnt5, Wnt7, Wnt9, Wnt11*, and *Wn16* are expressed in the pharyngeal ectoderm or mesoderm of gill slits (Geetha-Loganathan et al., 2009; Choe et al., 2013; Curtin et al., n.d.; Figure 5C)]. It is tempting to speculate, therefore, that the loss of many Wnt subfamilies (as well as the loss of the retinoic acid signaling Martí-Solans et al., 2016) in *O. dioica* could be related with major changes in the signaling requirements of this species for the formation of particular structures, such as the development of the gill slits.

### Function of the Maternal Wnt Signaling Pathway in *O. dioica*

The analysis of gene expression has shown that several Wnt signaling components of *O. dioica* (e.g., *Odi_Wnt11a*, *Odi_Fzd1/2/7 like, Odi_Fzd3/6a*, *Odi_Dvl*, *Odi_GSK3*, *Odi_*β*-catenin*…) are maternally expressed (Danks et al., 2013; Supplementary Figure 6). Among them, *Odi_Wnt11a* asymmetrically localizes in the posterior-most blastomeres during the early cleavages of development (Figure 3) resembling the expression of *Xenopus Wnt11* and ascidian *Wnt5* in the vegetal-posterior region (Ku and Melton, 1993; Sasakura et al., 1998).

This maternal expression of *Odi_Wnt11a* restricted to the posterior sub-cortical region of the posterior-most blastomeres suggests a role of *Wnt* signaling in A/P axis patterning (Figure 3). The localization of this mRNA correlates with the recently described “cytoplasm” that segregate to the posterior pole of the presumptive germ line blastomeres in *O. dioica*, called postplasm. In the *O. dioica* postplasm, maternal RNA and some morphological structures like the centrosome-attracting body (CAB) localize, reminding the postplasm of tunicate ascidians (Makabe and Nishida, 2012; Olsen et al., 2018). Interestingly, ascidian postplasm contains *Wnt5* that migrates to the posterior-most blastomeres to contribute to axis formation and cell fate determination (Prodon et al., 2007; Makabe and Nishida, 2012). Ascidian fertilized eggs injected with *Wnt5* morpholino cannot complete the gastrulation, and the asymmetric separation of the mRNAs necessary for the mesoderm endoderm segregation is also impaired (Nakamura et al., 2006; Takatori et al., 2010). When *Odi_Wnt11a* dsDNA were injected in the ovary of pre-mature *O. dioica* females, however, no defects in the cleavage and gastrulation was noticed, although a decrease in the amount of *Odi_Wnt11a* mRNA during the cleavage stages was observed (Figure 4E). It could be argued that the function of maternal *O. dioica* and ascidian Wnt signaling is different, or that functional inhibition by dsDNA was not strong enough to alter cleavage and gastrulation in *O. dioica* (Figure 4). Interestingly, it has been recently shown that knockdown of maternal β-catenin by dsDNA in *O. dioica* prevents the proper specification of the vegetal hemisphere (Omotezako et al., 2017). To fully understand the function of maternal Wnt signaling in *O. dioica* axis formation is, therefore, a challenging task that requires additional elaborate experiments such the generation of knockout lines for different Wnt genes by CRISPR that could overcome the technical limitations of lack of total penetrance of gene knockdowns.

## Data Availability Statement

The datasets presented in this study can be found in online repositories. The names of the repository/repositories and accession number(s) can be found in the article/Supplementary Material or in lab web file resources: https://evodevogenomics-unibarcelona.weebly.com/lab-resources–files.html.

## Author Contributions

JM-S, RA, and CC designed the experiments. JM-S, HG-M, and MD-G performed genome surveys and expression analyses. JM-S was responsible of the figures and tables, and performed evolutionary inferences. JM-S performed knockdown experiments under the supervision of TO and HN. JM-S, RA, and CC were responsible for writing the manuscript. RA and CC conceived and supervise the project. All authors commented on the manuscript and agreed to its final version.

## Conflict of Interest

The authors declare that the research was conducted in the absence of any commercial or financial relationships that could be construed as a potential conflict of interest.

## Footnotes

1 http://oikoarrays.biology.uiowa.edu/Oiko
