# Supplementary material for: Massive Gene Loss and Function Shuffling in Appendicularians Stretch the Boundaries of Chordate Wnt Family Evolution
Source: Front Cell Dev Biol. 2021 Jun 9;9:700827. doi: 10.3389/fcell.2021.700827 (PMC8220140; doi:10.3389/fcell.2021.700827)
Supplement: Supplementary file 1 [file Data_Sheet_1.pdf]

# *Supplementary Material*

## Table of Contents

1 Supplementary Methods....1
Preparation of PCR products for microinjection....1
Preparation of the mRNA for microinjection....1
Microinjection into the ovary....2
2 Supplementary Figures and Tables....3
Fig. S1. Evolution of appendicularian Wnt gene repertoire....3
Fig. S2. Wnt signaling components in *Oikopleura dioica*....5
Fig. S3. Expression patterns of *Oikopleura dioica* Wnt11b and Wnt11c genes during development....7
Fig. S4 Expression details of *Oikopleura dioica* Wnt genes during development....7
Fig. S5. Alignments of the *Wnt11a* DNAi target sequences....8
Fig. S6. Expression levels of Wnt signaling components in *Oikopleura dioica* across development and life time....8
Table S1. Putative *O. dioica* components of the Wnt signaling pathway....9
Table S2. Primer sequences used in this study....10
3 References for supplementary material....10

## 1 Supplementary Methods

**Preparation of PCR products for microinjection.** Each *Wnt* gene was amplified from a cDNA obtained from late hatchlings larvae with gene-specific primers (Table S2), cloned in the pCR4-TOPO vector (Invitrogen), transformed in TOP10 E. coli competent cells (Invitrogen), and sequenced using vector flanking primers. PCR products for microinjection were amplified using KOD plus (TOYOBO) and a specific inner pair of primers (Table S2). Single bands for each gene were obtained. Then, products were purified by phenol–chloroform and ethanol precipitation (adjusting the concentration of monovalent cations with sodium acetate at 0.3 M final concentration and pH 5.2). The DNA pellet was dissolved in 15 µL of water achieving a concentration of 0.5 µg/µL, approximately.

**Preparation of the mRNA for microinjection.** To generate the Lifeact-mCherry mRNA the pSD64TF-Lifeact-mCherry construct was used. Briefly, this construct is the Lifeact sequence amplified by PCR (forward primer, AAATTCTCGAGTCCACCATGGGTGTCGCAGATTTGAT; reverse primer, ACGTAGGGCCCTGGCGACCGGTGGATCC3) using pCMV Lifeact-TagRFP (Ibidi) as a template and subcloned into the XhoI/ApaI restriction sites of the pSD64TF-H2B-EGFP

vector, which include the SP6 polymerase promoter and the 5'- and 3'- UTR sequences of the β-globin mRNA of *Xenopus laevis*. (Omotezako et al., 2017, 2013). To generate the final Lifeact-mCherry, the EGFP sequence of the Lifeact-EGFP construct is replaced by the mCherry cDNA (Omotezako et al., 2013). For mRNA synthesis, the pSD64TF-Lifeact-mCherry plasmids was linearized with XbaI and used as template for in vitro transcription. Capped mRNA was synthesized with the SP6 of the mMESSAGE mMACHINE kit (Ambion) and polyadenylated with a Poly(A) Tailing Kit (Ambion). mRNA was purified by phenol-chloroform extraction and isopropanol precipitation, mRNA was dissolved in 20 µl of water achieving a concentration of 1.6 µg/µL, approximately.

**Microinjection into the ovary.** The ovary of *O. dioica* is a coenocyst where each pro-oocyte shares a common cytoplasm with the rest of the pro-oocytes (Ganot et al., 2007). Thus, any liquid injected into the ovary spreads to a large extent of the gonad and is incorporated, in a gradient manner, into 20–30% of spawned eggs. For injection, day 4 females, i. e. before oocytes became evident in the ovary, were selected, anesthetized with 0.015 % of Ethyl 3-aminobenzoate methanesulfonate salt (Sigma-Aldrich A5040) in seawater and set down on agar in a small drop of seawater. To immobilize the females the seawater was drained with a pipet. Then, using a micropipet mounted on a micromanipulator, the nucleic acids were injected into the ovary at the bottom of the gonad near to the junction of the trunk and tail. Approximately 1 nl of nucleic acid solution was injected with 1 mg/ml phenol red, to visualize the volume to be microinjected. mRNA encoding Lifeact-mCherry (0.6 µg/µL final concentration), as a marker of the nucleic acid incorporation, was co-injected with PCR products (0.2 µg/µL final concentration). After injection, each animal was placed separately in a six-well multititer plate coated with gelatin. 12 hours after injection, the animals spawned the eggs. Only eggs with mCherry fluorescence were fertilized and used for analysis, although there was a continuous distribution of different intensities from strongly fluorescent to non-fluorescent eggs.

## 2 Supplementary Figures and Tables

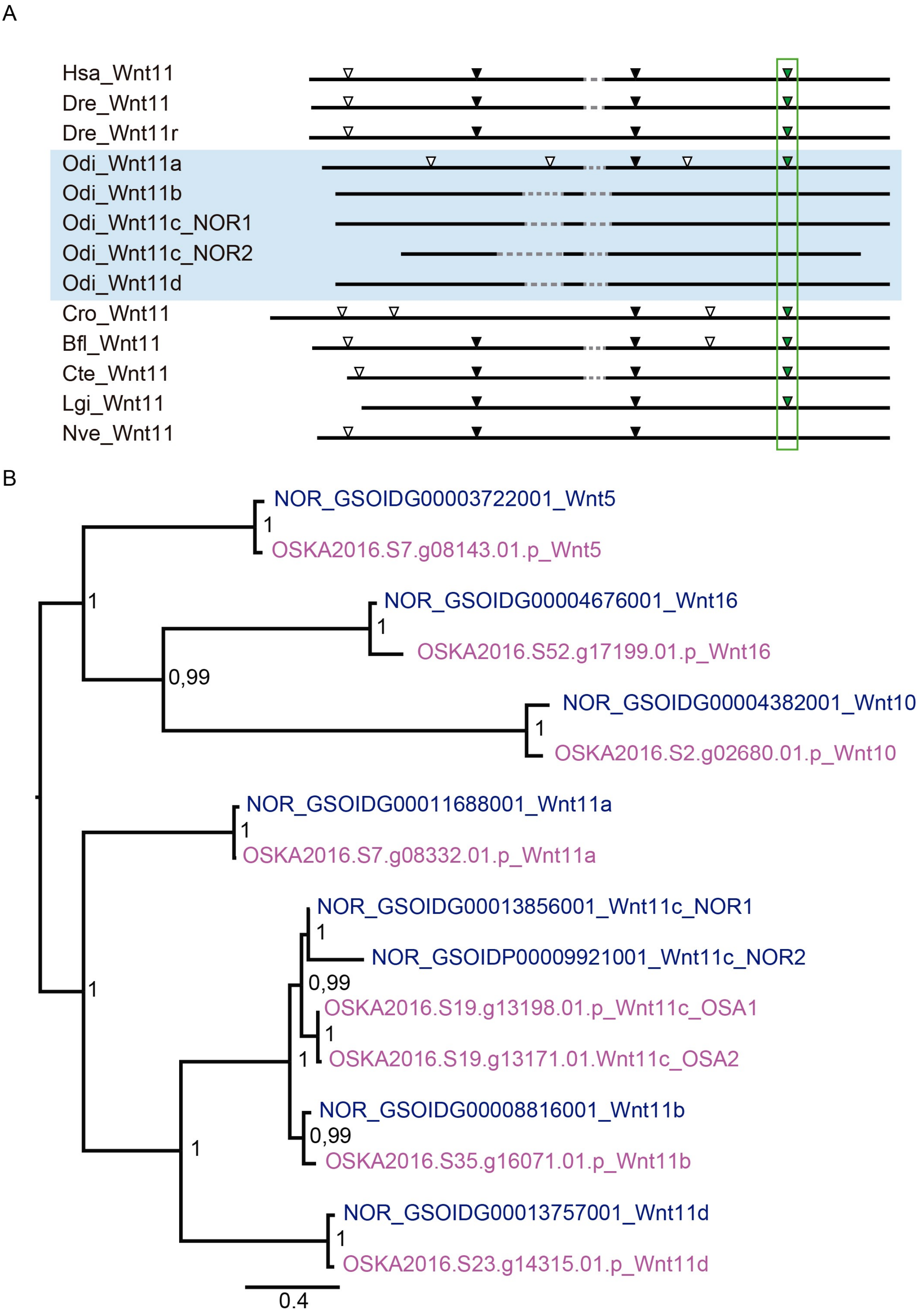

**Fig. S1. Evolution of appendicularian Wnt gene repertoire.** A. Schematic comparison of intron positions between human, ascidian and *O. dioica* genes. The CIWOG program (Wilkerson et al., 2009) from GECA package (Fawal et al., 2012) was used to compare the intron/exon organization of the different orthologous genes based on position and sequence conservation in the corresponding protein alignments, with no restrictions on the proportion of identical amino acids required to define a common intron. Black horizontal lines represent aligned sequences and arrowheads represent intron positions. Black arrowheads indicate introns conserved in all *Wnt* subfamilies across all metazoans, while white arrowheads indicate non-conserved introns. Green arrowheads indicate *Wnt11* subfamily specific intron shared between bilaterians. Red boxes indicate *O. dioica* conserved intron positions. Species abbreviations: Vertebrates: *Homo sapiens* (Hsa) and *Danio rerio* (Dre); Tunicates: *Ciona robusta* (Cro; formerly *Ciona intestinallis*) and *Oikopleura dioica* (Odi); Cephalochordates: *Branchiostoma floridae* (Bfl); Non-chordates species: annelid *Capitella teleta* (Cte), mollusk *Lottia gigantea* (Lgi) and cnidarian *Nematostella vectensis* (Nve). B. Maximum-likelihood tree, using a Bayesian Selection Criteria for the substitution model and an aBayes likelihood-based method for the calculation of node support, of Wnt sequences found in the genome assemblies of *O. dioica* specimens from Norway (in blue; NOR: http://oikoarrays.biology.uiowa.edu/Oiko) and Osaka (in pink; OSKA: http://www.aniseed.cnrs.fr). The tree corroborates that all Wnts genes of *O. dioica* belong to four Wnt subfamilies, and supports the presence of at least four paralogs in the Wnt11 subfamily (Wnt11a-d). The presence of two Wnt11c sequences in each populations (Wnt11c_NOR1-2 and Wnt11c_OSA1-2) in different clusters in the tree suggest the possibility that Wnt11c may have been independently duplicated in the two populations. The sequencing of the genome of other populations and further interpopulation comparisons will be needed to clarify the evolutionary history of Wnt11c and to discard that these sequences are not allelic variants that have been artifactually duplicated in the assembly of the genomes.

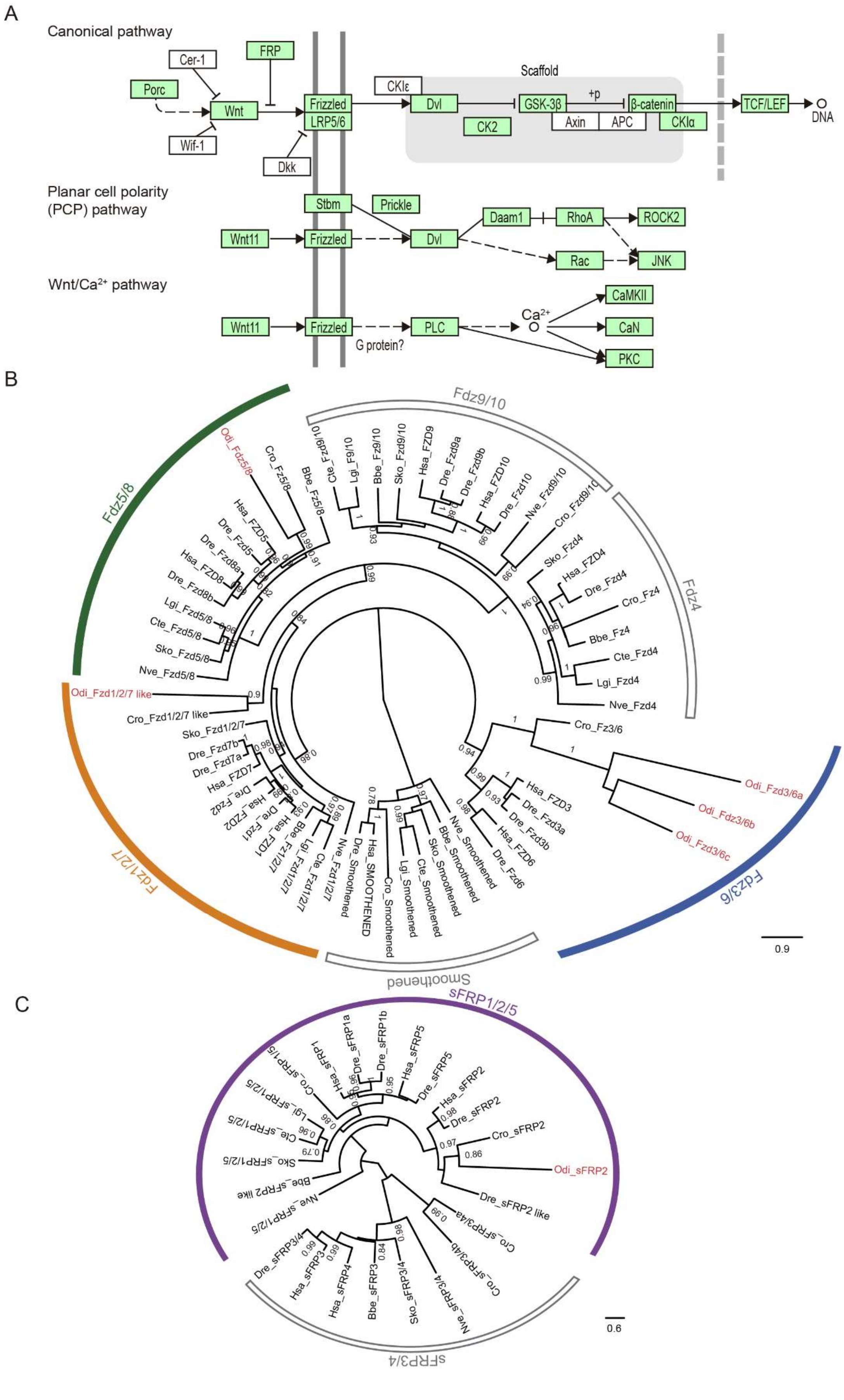
A
Canonical pathway
Cer-1
FRP
Porc
Wnt
Wif-1
Frizzled
LRP5/6
Dkk
CKIε
Scaffold
Dvl
GSK-3β
+p
β-catenin
CK2
Axin
APC
CKIα
TCF/LEF
DNA
Planar cell polarity (PCP) pathway
Stbm
Prickle
Wnt11
Frizzled
Dvl
Daam1
RhoA
ROCK2
Rac
JNK
Wnt/Ca2+ pathway
Wnt11
Frizzled
PLC
G protein?
Ca2+
CaMKII
CaN
PKC
B
Fdz5/8
Fdz9/10
Fdz4
Fdz3/6
Smoothened
Fdz1/2/7
Odi_Fdz5/8
Cro_Fz5/8
Bbe_Fz5/8
Hsa_FZD5
Dre_Fzd5
Dre_Fzd8a
Hsa_FZD8
Dre_Fzd8b
Lgi_Fzd5/8
Cte_Fzd5/8
Sko_Fzd5/8
Nve_Fzd5/8
Cte_Fzd9/10
Lgi_F9/10
Bbe_Fz9/10
Sko_Fzd9/10
Hsa_FZD9
Dre_Fzd9a
Dre_Fzd9b
Hsa_FZD10
Dre_Fzd10
Nve_Fzd9/10
Cro_Fzd9/10
Sko_Fzd4
Hsa_FZD4
Dre_Fzd4
Cro_Fz4
Bbe_Fz4
Cte_Fzd4
Lgi_Fzd4
Nve_Fzd4
Cro_Fz3/6
Odi_Fzd3/6a
Odi_Fz3/6b
Odi_Fzd3/6c
Hsa_FZD3
Dre_Fzd3a
Dre_Fzd3b
Hsa_FZD6
Dre_Fzd6
Nve_Smoothened
Bbe_Smoothened
Sko_Smoothened
Cte_Smoothened
Lgi_Smoothened
Cro_Smoothened
Hsa_SMOOTHENED
Dre_Smoothened
Odi_Fzd1/2/7 like
Cro_Fzd1/2/7 like
Sko_Fzd1/2/7
Dre_Fzd7b
Dre_Fzd7a
Hsa_FZD7
Nve_Fzd1/2/7
Cte_Fzd1/2/7
Lgi_Fzd1/2/7
Bbe_Fz1/2/7
Hsa_FZD1
0.9
C
sFRP1/2/5
sFRP3/4
Hsa_sFRP1
Dre_sFRP1a
Dre_sFRP1b
Hsa_sFRP5
Dre_sFRP5
Hsa_sFRP2
Dre_sFRP2
Cro_sFRP2
Odi_sFRP2
Dre_sFRP2 like
Cro_sFRP3/4a
Cro_sFRP3/4b
Nve_sFRP3/4
Sko_sFRP3/4
Bbe_sFRP3
Hsa_sFRP4
Hsa_sFRP3
Dre_sFRP3/4
Nve_sFRP1/2/5
Bbe_sFRP2 like
Sko_sFRP1/2/5
Cte_sFRP1/2/5
Lgi_sFRP1/2/5
Cro_sFRP1/5
0.6

**Fig. S2. Wnt signaling components in *Oikopleura dioica*.** A. KEGG Automatic Annotation server (KAAS) analysis of the *Wnt* pathway in *O. dioica*. BLAST program, bi-directional best hit (BBH) method and gene database of *Homo sapiens*; *Danio rerio*; *Ciona intestinalis*; *Branchiostoma floridae*; *Saccoglossus kowalevskii* and *Lottia gigantea* were used as parameters to obtain similar sequences for all *O. dioica*'s peptides and manual searches were performed to ensure the absence of the missing genes (Moriya et al., 2007). Green boxes highlight the assigned KEGG Orthology (KO), while white boxes refer to the failure in assignment to the specific KOs. B. The ML phylogenetic tree of the Fzd/Smoothened family showed the presence of three Fzd orthologues in *O. dioica* genome (red), corresponding to Fzr1/2/7, Fzd 3/6 (x3) and Fzd5/8, and the loss of Fzd4, Fzd9/10 and Smoothened orthologues. Smoothened family was used as outgroup to root the tree. C. The ML phylogenetic tree of the sFRP family showed that the single sFRP orthologue in *O. dioica* genome (red) is grouped with the sFRP2 genes. Values for the approximate likelihood-ratio test (aLRT) are only shown in nodes with support values greater than 0.75. Scale bar indicates amino-acid substitutions. The protein ID (GenBank accession number) used to elaborate the trees are as follows: Oikopleura dioica: Odi_Fdz1/2/7 like (GSOIDP00007729001) Odi_Fzd3/6a (GSOIDP00008718001), Odi_Fdz3/6b (GSOIDP00000671001), Odi_Fzd3/6c (GSOIDP00006033001), Odi_Fdz5/8 (GSOIDP00000721001), Odi_sFRP2 like (GSOIDP00005455001); Homo sapiens: Hsa_Fzd1 (Q9UP38), Hsa_Fzd2 (Q14332), Hsa_Fzd3 (Q9NPG1), Hsa_Fzd4 (Q9NPG1), Hsa_Fzd5 (Q13467), Hsa_Fzd6 (O60353), Hsa_Fzd7 (O75084), Hsa_Fzd8 (Q9H461), Hsa_Fzd9 (O00144), Hsa_Fzd10 (Q9ULW2), Hsa_Smoothened (Q99835); Hsa_sFRP1 (NP_003003), Hsa_sFRP2 (NP_003004), Hsa_sFRP3 (NP_001454), Hsa_sFRP4 (NP_003005), Hsa_sFRP5 (AAD25052); Danio rerio: Dre_Fzd1 (NP_001124086), Dre_Fzd2 (NP_571215), Dre_Fzd3a (NP_001036226), Dre_Fzd3b (NP_001074070), Dre_Fzd4 (NP_001292398), Dre_Fzd5 (NP_571209), Dre_Fzd6 (NP_956855), Dre_Fzd7a (NP_571214), Dre_Fzd7b (NP/739569), Dre_Fzd8a (NP_570993), Dre_Fzd8b (NP_571628), Dre_Fzd9a (XP_003198734), Dre_Fzd9b (NP_571586), Dre_Fzd10 (NP_570992), Dre_Smoothened (NP_571102), Dre_sFRP1a (NP_991148), Dre_sFRP1b (NP_001077040), Dre_sFRP2 (NP_001070852), Dre_sFRP2 like (XP_003200152), Dre_sFRP3/4 (NP_571018), Dre_sFRP5 (NP_571933); Ciona robusta: Cro_Fzd1/2/7 (NP_001071791), Ci_Fz3/6 (NP_001071723), Ci_Fz4 (XP_018667713), Cro_Fz5/8 (XP_009859677), Cro_Fzd9/10 (XP_002125798), Cro_Smoothened (XP_002125798), Cro_sFRP1/5 (NP_001071964), Cro_sFRP2 (NP_001072004), Cro_sFRP3/4a (NP_001071813), Cro_sFRP3/4b (NP_001071812); Branchiostoma belcheri: Bbe_Fzd1/2/7 (AHB53231), Bbe_Fzd4 (AHB53232), Bbe_Fzd5/8 (AHB53233), Bbe_Fzd9/10 (AHB53234), Bbe_Smoothened (XP_019641686), Bbe_sFRP2 like (AED89555), Bbe_sFRP3 (XP_019632159); Saccoglossus kowalevskii: Sko_Fzd1/2/7 (XP_006820151), Sko_Fzd4 (XP_002730495), Sko_Fzd5/8 (NP_001161547), Sko_Fzd9/10 (XP_006817188), Sko_Smoothened (XP_006817784) ; Capitella teleta: Cte_Fzd1/2/7 (ELT94235), Cte_Fzd4 (ELU03627), Cte_Fzd5/8 (ELT92800), Cte_Fzd9/10 (ELU07504), Cte_Smoothened (ELT97156), Cte_sFRP1/2/5 (ELU17320); Lottia gigantea: Lgi_Fzd1/2/7 (XP_009047666), Lgi_Fzd4 (XP_009050162), Lgi_Fzd5/8 (XP_009048933), Lgi_F9/10 (XP_009060024), Lgi_Smoothened (XP_009064048), Lgi_sFRP1/2/5 (XP_009051836); Nematostella vectensis: Nve_Fzd1/2/7 (XP_001647540), Nve_Fzd4 (XP_001622965), Nve_Fzd5/8 (XP_001634995), Nve_Fdz9/10 (XP_001630630), Nve_Smoothened (XP_001632182), Nve_sFRP1/2/5 (XP_001638620), Nve_sFRP3/4 (XP_001638660).

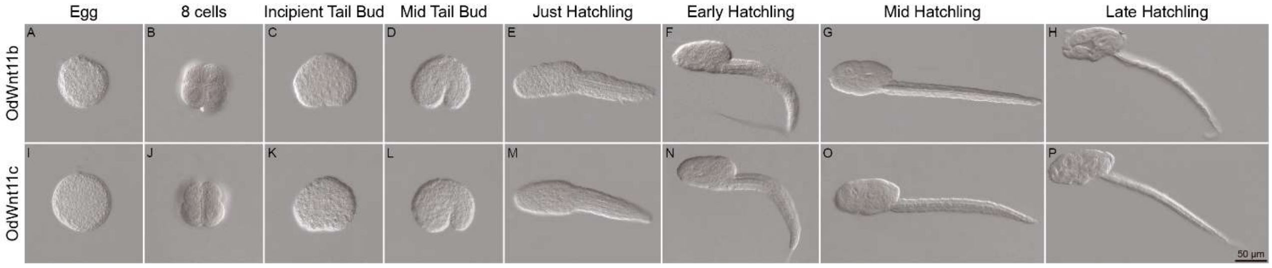


**Fig. S3. Expression patterns of *Oikopleura dioica* Wnt11b and Wnt11c genes during development.** Whole-mount in situ hybridization in *O. dioica* eggs (A and I), 8 cells embryos (B and J), incipient tail bud embryos (C and K), mid tail bud embryos (D and L), just hatchlings (E and M), early hatchlings (F and N), mid hatchlings (G and O) and late hatchlings (H and P). Each panel corresponds to left lateral view of the animal oriented anterior toward the left and dorsal toward the top. Scale bar = 50 µm

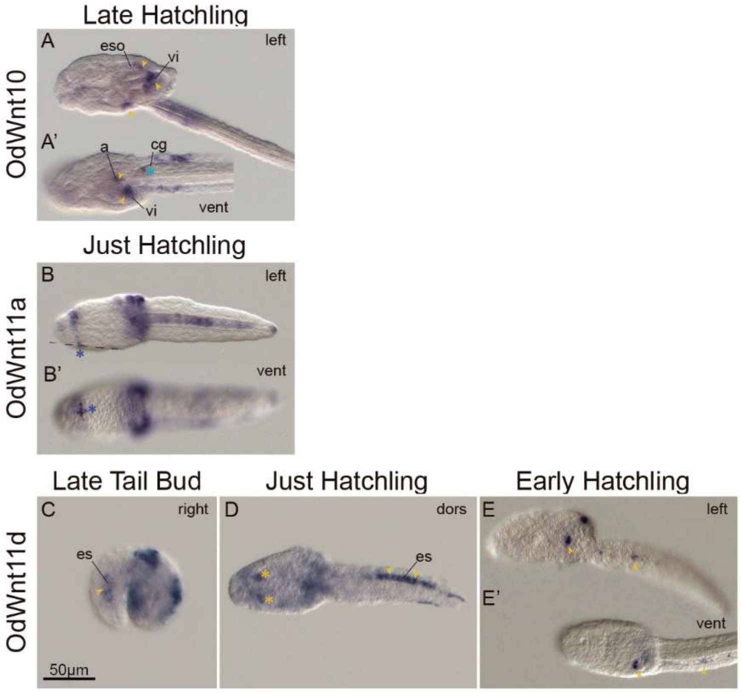


**Fig. S4 Expression details of *Oikopleura dioica* Wnt genes during development.** Whole-mount in situ hybridization in *O. dioica Wnt10, Wnt11a* and *Wnt11d* genes. Embryos were viewed from various directions indicated at the top. Yellow arrowheads point endodermal strand; yellow asterisks points endostyle; blue light asterisks point caudal ganglion; blue dark asterisks point oikoplastic epithelium; a, anus, cg, caudal ganglion; eso, esophagus; es, endodermal strand, vi, vertical intestine. Scale bar = 50 µm.

**OdWnt11a dsDNA #1**

92% Identity

```
Barcelona           1   ATGAAGATTTCAGTAACCCTTTTCTCTGGATTACTCTCTGCGGCATTCGGTATCGACTGG  60
Osaka (comp25579)   1   ..............C..A.......................A..C.....C.........  60

Barcelona          61   ATCGGCATGCACGGAAAGATGGTCGAAGACGATCTTTGCGATGGATTAAGCGA-CACGCT  119
Osaka (comp25579)  61   .....A.............................C.....C...........T..-...  119

Barcelona         120   GCAGTATCGTCTGTGTAGTAAATTTTCAAAAAATCGGAAAACGAAAGGATTTATGGAAGC  179
Osaka (comp25579) 120   ...............C...........G...G....A.......................  179

Barcelona         180   TATCCACACGGCCACAATCCAAACAAC  206
Osaka (comp25579) 180   C..........................  206
```

**OdWnt11a dsDNA #2**

91% Identity

```
Barcelona           1   GACTGTCACTCATATATGCAAATAACTAAAACTG--TATTAATTGTACATAAACAAAACC  58
Osaka (comp25579)   1   ...A..............................TT..............TC........  60

Barcelona          59   ATTATCTAAAAGCTTGGAAAATGAGAGAGCCTTTTTGCTATTCTCGGATTATTCTCGAGT  118
Osaka (comp25579)  61   ................A.............A.AAAA....................A...  120

Barcelona         119   TCAAACTGTATATTAACTCGCCCAGAGCGCAATTAACACATTGTACATGTTCGCTAGTAT  178
Osaka (comp25579) 121   .............A........T..T....C...C....TC...................  180

Barcelona         179   AACTTAACCACTTTATCATTGAACATTCTGCCCATATTTCCTACAGAAACTGTAAAAATA  238
Osaka (comp25579) 181   .............G.............T..............C..A....----------  230

Barcelona         239   TTTTAAACGTGAAATGAAAATTATCTTCAAAATATTTCATATTATGTATTCGATGTAAAA  298
Osaka (comp25579)       ------------------------------------------------------------

Barcelona         299   ATTGCATAAAAAGTACATTTTATCAGACCCAAATGGGCTCGAAAAGTTTCTTGTATGACT  359
Osaka (comp25579)       ------------------------------------------------------------

Barcelona         360   CCT  363
Osaka (comp25579)       ---
```

**Fig. S5. Alignments of the *Wnt11a* DNAi target sequences.** PCR products of the *Odi_Wnt11a* from the Barcelona's population were injected into embryos from Osaka. Alignments from the two populations and identities between sequences are shown. EST from the Osaka populations were shorter than Barcelona ones. Dots indicate identity.

| Pathway | Component | Oikopleura | Oocyte (Maternal transcripts) | 2-8 cells (Maternal transcripts) | 1HPF | Tailbud | Hatched | Early tadpole | Tailshift | Day 1 | Day 2 | Day 3 | Day 4 | Day 5 | Trunk | Testis | Ovary |
|---|---|---|---|---|---|---|---|---|---|---|---|---|---|---|---|---|---|
| Wnt activators | Wnt | GSOIDG00003722001 (Wnt5) | 0 | 0 | 0 | 0 | 0 | 1206 | 1522 | 626 | 505 | 418 | 0 | 0 | 0 | 0 | 0 |
| | | GSOIDG00004382001 (Wnt10) | 0 | 0 | 0 | 0 | 0 | 0 | 1027 | 409 | 228 | 216 | 0 | 0 | 0 | 0 | 0 |
| | | GSOIDG00004676001 (Wnt16) | 0 | 0 | 0 | 245 | 0 | 0 | 0 | 0 | 0 | 0 | 0 | 0 | 0 | 0 | 0 |
| | | GSOIDG00008816001 (Wnt11b) | 0 | 0 | 791 | 289 | 0 | 0 | 907 | 178 | 446 | 503 | 0 | 0 | 0 | 0 | 743 |
| | | GSOIDG00009921001 (Wnt11c_NOR2) | 0 | 0 | 0 | 0 | 0 | 0 | 0 | 0 | 0 | 0 | 0 | 0 | 0 | 0 | 0 |
| | | GSOIDG00011688001 (Wnt11a) | 1262 | 0 | 669 | 1451 | 1223 | 0 | 0 | 0 | 0 | 0 | 0 | 0 | 0 | 0 | 0 |
| | | GSOIDG00013757001 (Wnt11d) | 0 | 0 | 0 | 564 | 0 | 0 | 0 | 0 | 0 | 0 | 0 | 0 | 0 | 0 | 0 |
| | | GSOIDG00013856001 (Wnt11c_NOR1) | 0 | 0 | 0 | 0 | 0 | 0 | 0 | 0 | 0 | 0 | 0 | 0 | 0 | 0 | 0 |
| | Frizzled | GSOIDG00000671001 (Fzd3/6b) | 0 | 0 | 1743 | 10422 | 5071 | 6025 | 2428 | 580 | 618 | 527 | 0 | 0 | 0 | 0 | 0 |
| | | GSOIDG00000721001 (Fzd5/8) | 0 | 0 | 0 | 2707 | 2313 | 1466 | 1523 | 415 | 302 | 250 | 0 | 0 | 0 | 0 | 0 |
| | | GSOIDG00006033001/34001 (Fzd3/6c) | 0 | 0 | 0 | 0 | 0 | 0 | 532 | 588 | 0 | 0 | 0 | 0 | 0 | 0 | 0 |
| | | GSOIDG00007729001 ( Fzd1/2/7 like) | 11929 | 10416 | 15464 | 8889 | 3703 | 2039 | 2038 | 1011 | 1743 | 2251 | 1539 | 1539 | 4152 | 0 | 7039 |
| | | GSOIDG00008718001 (Fzd3/6a) | 4288 | 5532 | 2796 | 2974 | 2492 | 3484 | 697 | 0 | 0 | 0 | 0 | 0 | 0 | 0 | 0 |
| Wnt/β-catenin | LRP5/6 | GSOIDG00010381001 | 1849 | 1592 | 1463 | 1421 | 1107 | 2432 | 3398 | 877 | 1130 | 1474 | 776 | 645 | 0 | 1089 | 2404 |
| | Dishevelled | GSOIDG00009214001 | 4415 | 3253 | 4876 | 2814 | 1413 | 1025 | 1678 | 1058 | 1095 | 1017 | 929 | 675 | 0 | 0 | 1109 |
| | GSK3 | GSOIDG00003070001 | 7161 | 6319 | 10995 | 5708 | 3368 | 3111 | 4154 | 2349 | 3331 | 3593 | 3465 | 8293 | 2647 | 18778 | 6597 |
| | CKIα | GSOIDG00014955001 | 4990 | 4641 | 5973 | 4289 | 2463 | 2988 | 5602 | 4458 | 3935 | 4885 | 2684 | 4442 | 3482 | 8489 | 5950 |
| | CK2 | GSOIDG00002149001 | 7767 | 6258 | 8428 | 5201 | 3095 | 3174 | 4471 | 4765 | 5387 | 6374 | 5445 | 5971 | 3959 | 3262 | 7617 |
| | β-catenin | GSOIDG00004053001 | 5963 | 7108 | 8853 | 5922 | 5620 | 2763 | 1953 | 1025 | 1281 | 1502 | 1155 | 1392 | 0 | 0 | 3850 |
| | | GSOIDG00011813001 | 4002 | 4715 | 6376 | 4181 | 3820 | 3478 | 4532 | 3028 | 2248 | 2324 | 873 | 368 | 0 | 0 | 2235 |
| | TCF/LEF | GSOIDG00012371001 | 8510 | 8669 | 7360 | 12007 | 8549 | 6690 | 7585 | 3172 | 2603 | 3255 | 2363 | 2089 | 3548 | 0 | 4500 |
| Planar cell polarity | Daam1 | GSOIDG00009580001 | 6511 | 5882 | 8374 | 3076 | 1243 | 3115 | 3901 | 1243 | 1567 | 2033 | 1169 | 1279 | 0 | 0 | 4389 |
| | RhoA | GSOIDG00010718001 | 11722 | 11420 | 12994 | 8092 | 4883 | 11507 | 6393 | 6337 | 7762 | 7612 | 8206 | 7651 | 11072 | 2704 | 11681 |
| | ROCK2 | GSOIDG00000429001 | 8917 | 10096 | 14322 | 7968 | 3802 | 5348 | 10988 | 3987 | 3453 | 4277 | 3669 | 4462 | 2235 | 3042 | 10891 |
| | Rac | GSOIDG00007376001 | 11056 | 6601 | 12103 | 15105 | 10447 | 6069 | 6034 | 4329 | 5665 | 5207 | 5858 | 4166 | 5426 | 0 | 8078 |
| | JNK | GSOIDG00004808001 | 3677 | 2954 | 4518 | 1787 | 873 | 2190 | 2124 | 768 | 849 | 870 | 1004 | 971 | 0 | 697 | 2299 |
| | Strabismus | GSOIDG00009906001 | 13552 | 12385 | 16132 | 7480 | 6987 | 6044 | 3124 | 1082 | 1423 | 1420 | 1028 | 910 | 0 | 0 | 5025 |
| | Prickle | GSOIDG00017601001 | 2280 | 2039 | 4287 | 6077 | 3708 | 4289 | 3290 | 1987 | 1014 | 1058 | 686 | 529 | 0 | 0 | 1211 |
| $Ca^{2+}$ dependent | Phospholipase C | GSOIDG00016375001 | 3449 | 2994 | 3812 | 1317 | 651 | 1512 | 1436 | 895 | 787 | 1179 | 1302 | 953 | 0 | 0 | 2271 |
| | CaMKII | GSOIDG00001768001 | 0 | 0 | 0 | 1260 | 1179 | 3214 | 2344 | 1404 | 1103 | 970 | 727 | 649 | 0 | 0 | 0 |
| | Calcineurin | GSOIDG00003024001 | 0 | 0 | 0 | 486 | 859 | 1301 | 1029 | 319 | 247 | 267 | 0 | 0 | 0 | 0 | 0 |
| | Protein kinase C | GSOIDG00004710001 | 0 | 0 | 0 | 0 | 0 | 0 | 329 | 160 | 0 | 0 | 0 | 0 | 0 | 0 | 0 |
| Antagonists | sFRP | GSOIDG00005455001 | 578 | 0 | 895 | 649 | 801 | 0 | 0 | 0 | 0 | 0 | 0 | 0 | 0 | 0 | 0 |

**Fig. S6. Expression levels of Wnt signaling components in *Oikopleura dioica* across development and life time.** Gene expression values for *O. dioica Wnt* signaling components resulting from the KAAS analysis were extracted from the gene expression matrix of OikoBase (Danks et al., 2013). Oocyte and two to eight cell embryos are the two stages that encompass the maternal transcripts inherit by the embryos, while the following stages represents the transcriptional activity of the embryos; 1-hour post fertilization (hpf) and tailbud, larvae; hatched, early tadpole and tailshift, or adults; day 1, day 2, day 3, day 4, day 5, trunk, testis and ovary.

**Table S1. Putative *O. dioica* components of the Wnt signaling pathway.** E-values of bastp analysis of *O. dioica* proteins against non-redundant *H. sapiens* reference protein database.

| Component | Oikopleura | *H. sapiens* proteins with the highest E-values |
|---|---|---|
| Wnt/β-catenin | | |
| Wnt | GSOIDG00003722001 (*Wnt5*) | Wnt5 (3e-63; NP_001243034) |
| | GSOIDG00004382001 (*Wnt10*) | Wnt2 (7e-19; NP_003382.1) |
| | GSOIDG00004676001 (*Wnt16*) | Wnt4 (6e-35; NP_110388) |
| | GSOIDG00008816001 (*Wnt11b*) | Wnt4 (6e-35; NP_110388) |
| | GSOIDG00009921001 (*Wnt11c_NOR2*) | Wnt7 (3e-17; XP_011528668.1) |
| | GSOIDG00011688001 (*Wnt11a*) | Wnt4 (2e-51; NP_110388) |
| | GSOIDG00013757001 (*Wnt11d*) | Wnt4 (3e-35; NP_110388) |
| | GSOIDG00013856001 (*Wnt11c_NOR1*) | Wnt4 (2e-34; NP_110388) |
| Frizzled | GSOIDG00000671001 (*Fzd3/6b*) | Fdz7 (1e-64; NP_001158088) |
| | GSOIDG00000721001 (*Fzd5/8*) | Fzd5 (2e-153; NP_003459) |
| | GSOIDG00006033001/34001 (*Fzd3/6c*) | Fzd7 (1e-66; NP_003498) |
| | GSOIDG00007729001 (*Fzd1/2/7* like) | Fdz2 (8e-129 NP_001457) |
| | GSOIDG00008718001 (*Fzd3/6a*) | Fzd6 (4e-25; NP_003497) |
| LRP5/6 | GSOIDP00010381001 | LRP5 (1e-60; XP_011543332) |
| Dishevelled | GSOIDG00009214001 | Dvl (3e-129; NP_004414) |
| GSK3 | GSOIDG00003070001 | GSK-3β (0.0; NP_001139628) |
| APC | Absent | |
| Axin | Absent | |
| CKIα | GSOIDG00014955001 | CKIα (0.0; NP_001883) |
| CKIε | Absent | |
| CK2 | GSOIDG00002149001 | CK2 (0.0; NP_001886) |
| β-catenin | GSOIDG00004053001 | CTNNB1 (1e-108; NP_001317658) |
| | GSOIDG00011813001 | CTNNB1 (0.0; NP_001317658) |
| TCF/LEF | GSOIDG00012371001 | TCF7L2 (2e-52; XP_011538413) |
| Planar cell polarity | | |
| Daam1 | GSOIDG00009580001 | DAAM2 (0.0; NP_056160) |
| RhoA | GSOIDG00010718001 | RhoA (3e-102; NP_001655) |

| | | |
|---|---|---|
| ROCK2 | GSOIDG00000429001 | ROCK1 (1e-37; NP_005397) |
| Rac | GSOIDG00007376001 | RAC1 (5e-114; NP_008839) |
| JNK | GSOIDG00004808001 | JNK3 (0.0; XP_016863916) |
| Strabismus/van Gogh | GSOIDG00009906001 | VANGL1 (2e-118; NP_001165882) |
| Prickle | GSOIDG00017601001 | PRICKLE2 (4e-122; XP_011531742) |
| Ca2+ dependent | | |
| Phospholipase C | GSOIDG00016375001 | PLCB4 (1e-155; XP_011527556) |
| CaMKII | GSOIDG00001768001 | CaMKII (0.0; XP_006714393) |
| Calcineurin | GSOIDG00003024001 | CALN (0.0; NP_001124163) |
| Protein kinase C | GSOIDG00004710001 | PKC (0.0; NP_997700) |
| Antagonists | | |
| Dickkopf | Absent | |
| WIF | Absent | |
| Cerberus | Absent | |
| sFRP | GSOIDG00005455001 | sFRP2 (1e-27; NP_003004) |

**Table S2. Primer sequences used in this study.**

| Primers for riboprobe synthesis | | |
|---|---|---|
| | Forward Sequence (5'->3') | Reverse Sequence (5'->3') |
| Wnt5 | ATGGCGTCAAAAAACGCTCTTCAAG | GCGAGGCTATTTGCATTTGTAGATTTT |
| Wnt10 | ATGTCGAAGGAAACGAAAAATGAG | CTAGGTACAAACGCCGTATGTTAC |
| Wnt11a | ATGAAGATTTCAGTCACCCTTTTCTCTG | GTTATTTGCATATATGAGTGACAGTCG |
| Wnt11b | ATGAGAATCTCCAACATTCTCTTCGC | GATTATCGGCATGTGCTTGTGG |
| Wnt11c | ATGAAAATCTTTAACGTCCTCTTTGC | TCAACGGCAGGTGCTCGTCG |
| Wnt11d | ATGAAAATAACTTTCTTTTCTCTTTTCGC | GGTTTAACGGCAAGAATGTGAAAT |
| Wnt16 | ATGGCAATGCAACAATGTGAAACTG | GCTTATATACAGTAGTACTTCGTCT |
| Primers for dsDNA injection | | |
| Wnt11a dsDNA #1 | ATGAAGATTTCAGTCACCCTTTTCTCTG | GTTGTTTGGATTGTGGCCGTGTG |
| Wnt11a dsDNA #2 | GTATGTATGCGGCCGCGACTGTCACTCATATAT GCAAATAA | GAGGAGTCATACAAGAAACTTTTCG |

## 3 References for supplementary material

1. Danks G, Campsteijn C, Parida M, Butcher S, Doddapaneni H, Fu B, Petrin R, Metpally R, Lenhard B, Wincker P, Chourrout D, Thompson EM, Manak JR. (2013) OikoBase: A genomics and developmental transcriptomics resource for the urochordate Oikopleura dioica. *Nucleic Acids Res* 41(D1):1–9.

2. Fawal N, Savelli B, Dunand C, Mathe C. (2012). GECA: a fast tool for gene evolution and conservation analysis in eukaryotic protein families. Bioinformatics 28:1398–1399.
3. Ganot, P., Bouquet, J. M., Kallesøe, T. and Thompson, E. M. (2007). The Oikopleura coenocyst, a unique chordate germ cell permitting rapid, extensive modulation of oocyte production. *Dev. Biol.* 302, 591–600.
4. Moriya Y, Itoh M, Okuda S, Yoshizawa AC, Kanehisa M (2007) KAAS: an automatic genome annotation and pathway reconstruction server. *Nucleic Acids Res* 35(Web Server issue):W182-5.
5. Omotezako T, Nishino A, Onuma TA, Nishida H (2013) RNA interference in the appendicularian Oikopleura dioica reveals the function of the Brachyury gene. *Dev Genes Evol* 223(4):261–267.
6. Omotezako T, Matsuo M, Onuma TA, Nishida H (2017) DNA interference mediated screening of maternal factors in the chordate Oikopleura dioica. *Nat Publ Gr* (March):1–10.
7. WilkersonMD, Ru Y, Brendel VP. (2009). Common introns within orthologous genes: software and application to plants. Brief Bioinform. 10:631–644.
